# A statistical approach to knot confinement via persistent homology

**DOI:** 10.1098/rspa.2021.0709

**Published:** 2022-05-25

**Authors:** Daniele Celoria, Barbara I. Mahler

**Affiliations:** Mathematical Institute, University of Oxford, Radcliffe Observatory, Andrew Wiles Building, Woodstock Rd, Oxford OX2 6GG, UK

**Keywords:** knots, topological data analysis, persistent homology

## Abstract

In this paper, we study how randomly generated knots occupy a volume of space using topological methods. To this end, we consider the evolution of the first homology of an immersed metric neighbourhood of a knot’s embedding for growing radii. Specifically, we extract features from the persistent homology (PH) of the Vietoris–Rips complexes built from point clouds associated with knots. Statistical analysis of our data shows the existence of increasing correlations between geometric quantities associated with the embedding and PH-based features, as a function of the knots’ lengths. We further study the variation of these correlations for different knot types. Finally, this framework also allows us to define a simple notion of deviation from ideal configurations of knots.

## 1 Introduction

In this paper, we consider the following question: Does the topology of a random knot, i.e. its knot type, influence how it ‘occupies’ space? More specifically, do more complicated knots tend to be more compact or loose with respect to their simpler counterparts of the same length? Similar ideas have previously been considered in e.g. [1–4], often with the main goal of understanding the mechanism of DNA, or, more generally, polymer packing in a confined volume. Prominent instances of such studies are concerned with tight DNA packing in viral capsids [5–8] and DNA packing in cells (e.g. [9,10] and the review [11]). We refer to the comprehensive survey [12] for further references and several related notions.

Our main tool to address these matters is persistent homology (PH) [13,14]; this is a relatively new technique in topological data analysis, commonly used to detect insightful topological and geometric features of point clouds. Roughly speaking, PH associates a filtered simplicial complex to a point cloud. The filtered homology groups of these chain complexes often capture subtle properties of the point cloud [15].

We start by generating random piecewise linear (PL) knots with prescribed length and/or topology. We then create a point cloud for each knot K by linearly interpolating between the endpoints of the PL curve, and we compute the PH of its Vietoris–Rips filtration in dimension 1. Intuitively, we use PH to examine the changes in topology occurring in a metric neighbourhood of a random knot, when the radius of this neighbourhood varies in $R_{\geq 0}$.

We extract several features from the obtained PH and quantify the variation in the correlation between these features and either the volume ‘occupied’ by the knot or the average crossing number (ACN) for increasing lengths and for all knot types with up to six crossings. The most prominent feature we extract from PH is I(K), the integral of the Betti curve of a persistence diagram, which might be of independent interest.

Note that unlike previous approaches, such as those outlined in [12, §8], we do not prescribe the geometry of the confined volume, but rather work backwards, by first generating the knots and only afterwards analysing their relationship with the minimal volume that contains them. More precisely, we consider different kinds of measures for the space ‘filled in’ by a knot: the volume of the circumscribing sphere, i.e. the volume of the smallest sphere that encloses the knot, the volume of the convex hull determined by the knot and the radius of gyration. This last quantity is often used as a meaningful and computationally convenient measure of compactness of proteins and polymers (e.g. [12,16–19]). We remark that, in the case of proteins, compactness is defined as the ratio of the accessible surface area of a protein to the surface area of the sphere of the same volume. We instead consider a more intuitive and geometric notion of compactness.

Most prominently, we show the existence of an inverse correlation between the integral I(K) and the various notions of volume occupied by K mentioned above, as well as a direct correlation between I(K) and the ACN. The magnitude of these correlations increases for increasing knot lengths. Furthermore, we find that these correlations appear to differentiate between different knot types. Peculiarly, the intensity of the computed correlations does not appear to be directly related to classical measures of a knot’s complexity, such as the minimal crossing number.

To better appreciate these results, especially the resulting subdivision into knot types, we also compute the average Betti curve for each knot type considered, as well as their integrals. We observe an almost perfectly linear relation between the average integrals of the Betti curves and the knot lengths; the same holds for the average maxima of the Betti curves, which are in turn correlated to the average number of shallow angles in the embeddings. Indeed, these relations show a clear divide among the considered knot types.

We then turn to the related concept of *ideal knot* embeddings. These are special embeddings of knots, whose study was pioneered by Stasiak & Katritch [20]. Their geometry is particularly simple, in that they minimize the length of a rope (having unit diameter) that is needed to tie a specific knot. We show how the PH framework developed here can be used to define a simple numerical measure of how ‘far away’ a given knot is from an ideal embedding.

We make the concepts mentioned in the introduction rigorous in §2, and give a basic overview of the techniques we use in §3. We then detail how we generated our data in §4, and present the results in §5. Finally, in §6, we define the aforementioned deviation from ideal knot embeddings.

## 2. Knot theory

We call a *knot* K the image of a smooth embedding of $S^{1}$ in $S^{3}$, and reserve the notation K to denote knot types. We refer to [21] for basic definitions in knot theory (see also [22, Ch. 8]). In what follows, we relax the smoothness condition to allow the approximation of a smooth embedding by equilateral polygons. These curves will be referred to as PL knot embeddings.

The *length* ℓ(K) of a knot K is the usual Euclidean length of K. When considering the length in the PL case, we always require all segments composing a given polygonal knot to be of equal length; we take each segment of unit length, so that ℓ(K) coincides with the number of edges used.

**Figure 1.**
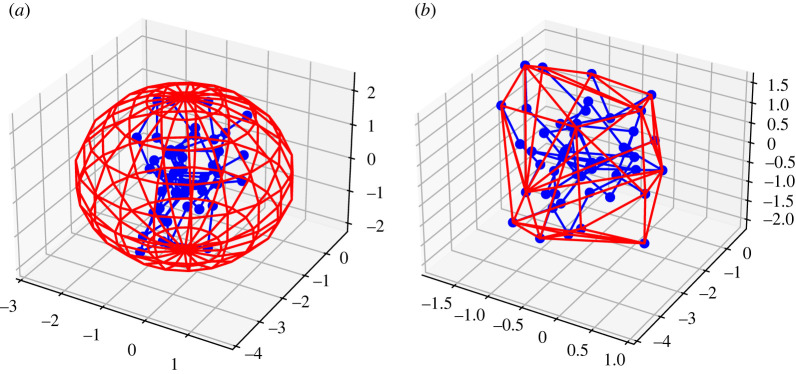
(*a*, *b*) In red, a circumscribing sphere and convex hull for the blue PL trefoil knot with 50 edges. In this example, the sphere has volume approximately 50, while the convex hull’s volume is approximately 14.

We are interested in investigating how efficiently a given embedding can occupy a volume; we therefore consider different kinds of measures of compactness for a knot embedding. In increasing order of accuracy, for each K we compute the volume of the minimal sphere and the convex hull surrounding K (figure 1). We also take into consideration the radius of gyration Rg of a PL-embedded knot.

**Figure 2.**
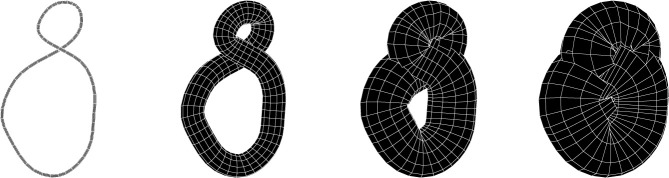
The evolution of the neighbourhood $\nu_{t}(K)$ for an eight-shaped unknot. The last stage on the right is homeomorphic to a 3-ball. The image was generated using KnotPlot [23].

We denote by $\nu_{t}(K)$ the metric neighbourhood of K of radius t>0. We obtain this by considering the union of the radius t discs contained in the affine planes $P_{p}+p$ that are centred at the points p in the image of the embedding, and that are orthogonal to the embedding. We crucially point out that in what follows we will not necessarily only consider regular (i.e. non-self-intersecting) neighbourhoods (figure 2).

The *injectivity radius* IR(K) of K (see [24]) is the supremum among all radii t≥0 for which the tubular neighbourhood $\nu_{t}(K)$ is regular. In other words, IR(K) is the smallest value of the neighbourhood’s radius such that $\nu_{t}(K)$ comes into contact with itself. Intuitively, if we regard the given knot K as being made of rope, IR(K) represents the maximal radius that the rope can have while being knotted in the ‘shape’ of K.

The *length over diameter ratio* of K is the quotient

$$L/D(K)=\frac{\ell (K)}{2\text{IR}(K)},$$

between the length of K and twice the injectivity radius. The ratio L/D was introduced and has been extensively studied by Stasiak & Katritch [20] (the inverse of this quantity is also known as the *thickness* of K [24]), due to its relation with the notion—also introduced by Stasiak—of ideal knot.

An *ideal knot* (see the monograph [20]) is an embedded knot that minimizes the L/D ratio within its knot type. Note that *a priori* there might be more than one ideal representative of a given knot type. One interpretation of L/D for an ideal knot K is as the minimal length needed to tie a knot of the knot type of K with a rope that has diameter 1.

We will also use other quantities that can be associated with PL knot embeddings: a discrete analogue of torsion and curvature, and the ACN. This latter quantity is defined as the integral of the function $S^{2}⟶N$ associating to a point p on the unit sphere the crossing number of the diagram obtained by projecting K onto the plane tangent to the sphere at p (strictly speaking, we should also renormalize by dividing by 4π and restrict to projections with at most double points as singularities).

We have just seen that the notion of injectivity radius is crucial for the definition of several knot properties. One of the key technical aspects of this paper is the use of a straightforward generalization of IR, and that PH can be effectively used to compute it. Given a knot K, consider the neighbourhood $\nu_{t}(K)$ for t∈[0,∞[. For small enough t, the homology of the embedded neighbourhood is of rank 1 in dimension 1. The topology changes as soon as we get to t=IR(K), where (generically) the rank of the homology of $\nu_{t}(K)$ increases by one. Similarly, for increasing t, we can keep track of all the times t where the topology of $\nu_{t}(K)$ changes. Note that for values of t greater than RS(K), the radius of the circumscribing sphere, ${\text{H}}_{1}(\nu_{t}(K))$ vanishes, and the only non-trivial homology is of rank 1 in degree 0.

We can now introduce the Betti curve for the first homology, which is one of the main objects we will consider in what follows.

**Definition 2.1.**

Call the (first) *Betti curve* of a knot K the integer valued function

$$\beta_{1}(K):R_{\geq 0}⟶N,$$

defined as $t\mapsto \text{rk}({\text{H}}_{1}(\nu_{t}(K)))$.

It follows from the previous discussion that $\beta_{1}(K)$ is 1 for small values of t and becomes definitely 0 for t≫0.

An interesting property of Betti curves is that (just as with persistent landscapes [25]) we can add them and take averages; we will take advantage of this fact in §5.

Note that if K presents some small-scale configuration (such as the smaller twirl in figure 2), then, after increasing the radius more than a certain threshold, its contribution to $\text{rk}({\text{H}}_{1}(\nu_{t}(K)))$ vanishes (after the neighbourhood engulfs the small-scale configuration).

We will argue shortly that PH can be used to closely approximate $\beta_{1}(K)$. In fact, by considering distributions of points closely approximating the embedding K and increasing in density, we get increasingly better approximations of $\beta_{1}$.

**Example 2.2.**

**Figure 3.**
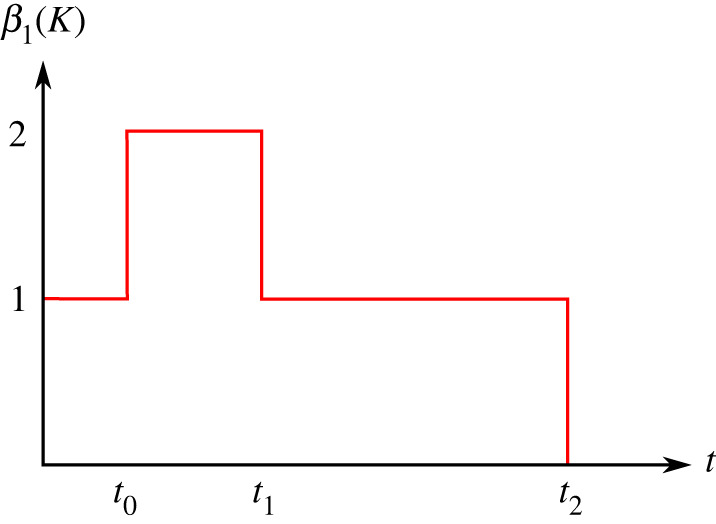
A schematic of the function $\beta_{1}(K)$ for the unknotted embedding from figure 2. The values $t_{i}$ mark the times of t where the topology of $\nu_{t}(K)$ changes. (Online version in colour.)

Consider the planar standard embedding ◯ of $S^{1}$ in $R^{3}$, with radius R. Then $\beta_{1}(◯)\equiv 1$ on [0,R[, and 0 on [R,∞[. For the rather simple unknotted embedding in figure 2, the Betti curve resembles that of figure 3.

**Remark 2.3.**

**Figure 4.**
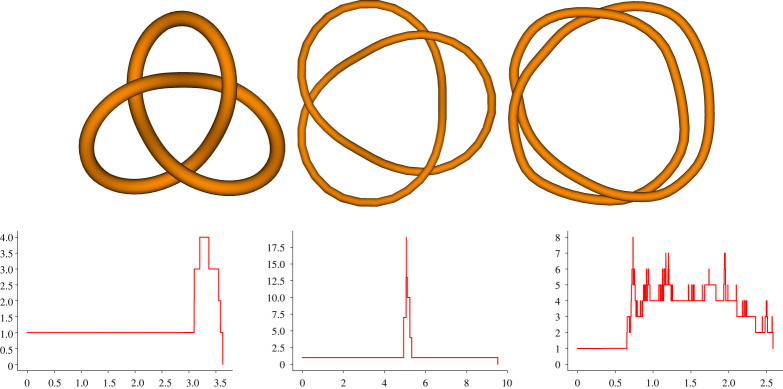
Three different embeddings of the trefoil, and their corresponding (approximated) Betti curves. From left to right: a trefoil embedding close to its ideal configuration, a trefoil lying on a standard torus whose longitude is longer than its meridian, and a trefoil close to being planar. The images in the top panel were obtained with Knotplot [23]. (Online version in colour.)

If K is an ideal configuration [20] for the knot type K, then we expect $\beta_{1}(K)(t)$ to be 1 for $t\in [0,\frac{1}{2}[$ (since the injectivity radius is by definition $\frac{1}{2}$), and then jump to a large number m(K) immediately after (see the left part of figure 4). The number m(K) is related to the number of self-tangencies of the ropelength minimizer embedding considered. We can use this to provide a measure of the ‘closeness’ between a given embedding and ideal configurations. We will define such a measure in §6.

## 3. PH

**Figure 5.**
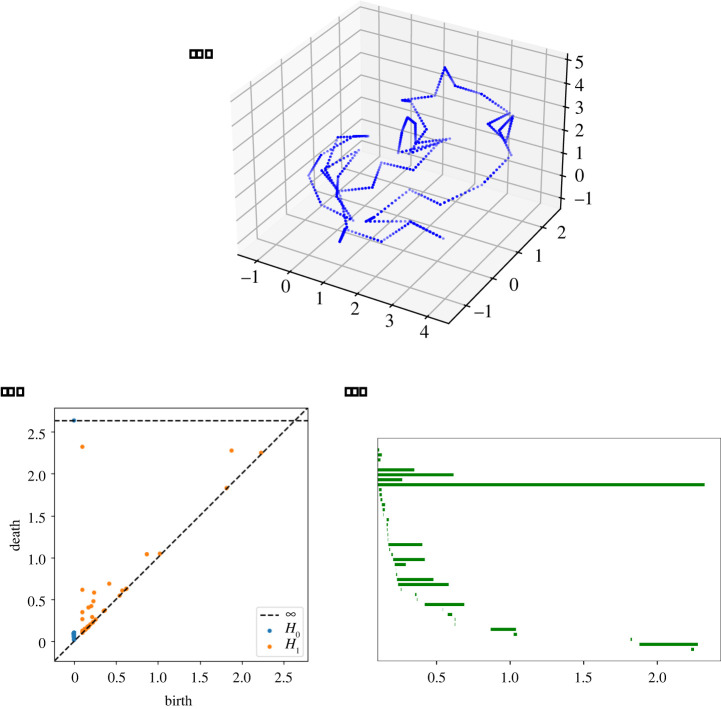
In (*a*), a PL knot embedding of the trefoil with 50 edges. Each edge is replaced with 10 equidistant dots to create a point cloud (see also figure 6). In (*b*,*c*), two different ways to visualize the persistence diagram of this point cloud (in the barcode on the right, we are only considering homology in dimension one). In the persistence diagram on (*b*), each point corresponds to a homology and its placement indicates the value of t at which the class appears (birth) and the value of t at which the class disappears (death). In the barcode on (*c*), homology classes are represented by bars that start and end at the birth and death value of t, respectively.

PH [15] is an algebraic tool for detecting topological and geometric properties of a space at different resolutions. The input for PH is a nested sequence of simplicial complexes, called a *filtration*. There are various ways to build a filtration on point-cloud data. Commonly used constructions are made from Čech complexes, Vietoris–Rips complexes or α-complexes. Roughly speaking, PH captures the evolution of the homology of the filtered complex as it grows through the filtration. In particular, it keeps track of how homology classes appear and disappear, and this information can be represented in a *persistence diagram* or *barcode* (figure 5). The Čech complex built on a point cloud P for a given radius t≥0 is the simplicial complex whose 0-simplices are the points in P, and whose higher dimensional simplices are subsets of points in P whose closed t-balls have non-empty intersection. This complex has the same homotopy type as the union of the closed t-balls centred in the points in P, by the *Nerve Lemma* (e.g. §III.2 of [15]). The Čech filtration on P is the filtration consisting of the Čech complexes on P for growing radii t≥0, and it gives a topologically faithful representation of the gradual thickening of the underlying space if P is a sufficiently dense and uniform sample of the space.

However, for large data, it can be computationally intensive—and hence impractical—to build a Čech filtration [26]. Therefore, in practice, one may want to instead work with the Vietoris–Rips filtration, which depends only on pairwise distances between points and can therefore be computed much more efficiently. The Vietoris–Rips complex built on a point cloud P for a given radius t≥0 is the simplicial complex whose 0-simplices are the point in P, whose 1-simplices are the pairs of points in P that are within a distance of 2t from each other, and whose higher dimensional simplices are the cliques of its 1-simplices. The Vietoris–Rips filtration on P is the sequence of Vietoris–Rips complexes for growing radii t≥0, and it is a good approximation to the Čech filtration in Euclidean space [27].

We use the Vietoris–Rips filtration on a point cloud P(K) constructed from a PL knot embedding K to approximate its metric neighbourhood for growing radii.

We consider several features of the barcode corresponding to the Vietoris–Rips filtration on P(K); the first are simply the number of bars and the length of the longest bar in the barcode in degree 1 of the PH of P(K). These are denoted by #B(K) and M(K), respectively. Furthermore, similarly to the Betti curve of a growing neighbourhood of a knot (see definition 2.1), we can define a Betti curve on the rank of the homology of the Vietoris–Rips filtration on a point cloud. We compute the integrals of such Betti curves $\int_{0}^{\infty }\beta_{1}(P(K))\;\text{d}t$ by summing the lengths of all bars in the corresponding barcode. We denote the integral of the Betti curve corresponding to the Vietoris–Rips filtration on a point cloud sampled from a knot K by I(K). We will see in §5 that the integral I(K) as defined here constitutes a good approximation (with a few caveats, as explained in figure 9) of the integral of the Betti curve from definition 2.1.

## 4. The data

In this section, we describe the samples we study, as well as the methodologies used to generate and analyse them.

All the random knots in our datasets are generated using the excellent Python-based Topoly [28]. The same program is also used to compute the HOMFLY polynomial, in order to determine the knot types of the PL curves considered whenever required. The random generation function provided by Topoly is based upon the algorithm by Cantarella *et al.* [29]; this algorithm guarantees uniformity of sampling within the space of equilateral polygonal curves with a fixed number of segments. However, the authors are not aware of a uniform way of sampling polygonal representatives within a given knot type and length. Our sampling—for a fixed knot type—is therefore carried out by first generating a large population of polygons, and then only keeping those with the required knot type.

We use pyknotid [30] to compute ACN. We use custom-made programs to compute the radius of gyration, curvature, torsion and volume of the smallest enclosing sphere of a PL-embedded knot, and we use SciPy’s [31] built-in function to compute the volume of its convex hull.

We produce two qualitatively different datasets. For the first dataset, we generate $10^{4}$ random knots for each length from 10 to 100 (in steps of 10). We then compute the volume of the minimal enclosing sphere and that of the convex hull inscribing each knot, as well as the curvature, torsion, ACN and radius of gyration.

For the second dataset, we sample $10^{3}$ knots for each prime knot type with up to six crossings, for lengths between 50 and 200 (in increments of 50); we also include $10^{3}$ samples of knots whose knot types do not belong to the previous categories (so whose minimal crossing number is greater than or equal to 7), and we will refer to these as ‘unknown’ in what follows.

**Figure 6.**
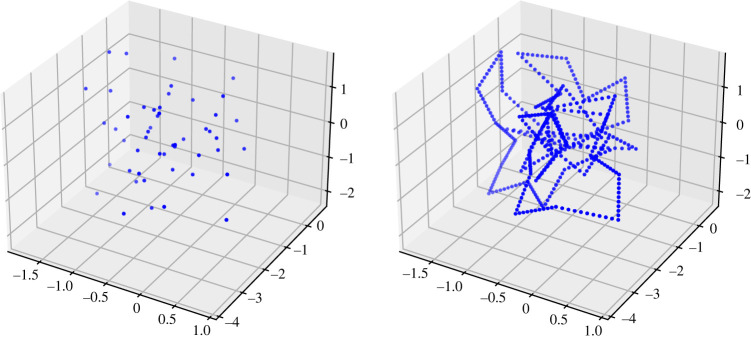
A point cloud describing the endpoints of a PL embedding K of the trefoil with 50 edges, and the point cloud P(K) we consider, obtained by interpolating the unit length edges with 10 further equidistanced points. (Online version in colour.)

We then compute the barcodes for Vietoris–Rips filtrations associated with the knots as explained below using the efficient program Ripser [32]. As our purpose is to approximate the topology of the neighbourhoods of our generated embeddings as closely as possible, we do not simply compute the PH of the Vietoris–Rips filtrations on the endpoints of the sampled PL curves. Instead, we interpolate the endpoints of the unit segments of each embedding with 10 equidistant points, and compute the Vietoris–Rips filtration on the resulting point cloud, which we denote by P(K) (figure 6). Using 10 points per linear segment gives a dense enough sample of the embedding to yield a Vietoris–Rips filtration that approximates the growing tubular neighbourhood closely for most knots. For practical reasons, we restrict our considerations to the first homology groups. It is likely that higher homology groups do also retain useful information on the geometric structure of such embeddings.

In order to be able to find meaningful correlations between geometric aspects—such as enveloping volumes, curvature, torsion and the ACN—and PH, we consider the features extracted from the barcodes described in the previous section. Note that, in the case at hand, the support of $\beta_{1}(K)$ is contained in the interval [0,RS(K)], where again RS(K) is the diameter of the minimal sphere enclosing the embedding. Indeed, for t≥RS(K) the neighbourhood $\nu_{t}(K)$ is topologically a 3-ball. This readily implies that I(K) is well defined, i.e. it is a proper integral.

The correlations displayed in the next section were computed using SciPy’s statistics module. All the programs we used to generate the data, as well as the data itself, are available on the first author’s GitHub page [33].

## 5. Results

In this section, we collect the results of the computations detailed above. More precisely, we show that the correlation between the integral of $\beta_{1}$ and the volume occupied by a knot becomes increasingly negative as the knot’s length increases. The magnitude of these correlations is especially large in the case of the volume of the convex hull. This confirms the intuitive fact that, for long embeddings, being geometrically complex implies being spatially compact. Furthermore, we quantify how considering increasingly complex topologies (i.e. progressively complex knot types) influences this inverse correlation.

Similarly, we find a growing correlation between I(K) and the ACN (left panel of figure 12). This is not surprising, as both can be thought of as being measures of the geometric complexity of the embedding; it is however interesting to observe the phenomena displayed in figure 12*b*, where this correlation’s behaviour is split among the different knot types. Here, we observe that, rather unexpectedly, the values of the plots do not appear to be monotonically related to the complexity measure on knot types given by the minimal crossing number. Indeed, the ‘unknown’ category has values which are larger than most other knot types (in the range of lengths considered). At the same time, the unknots’ correlations appear to be considerably larger.

**Figure 7.**
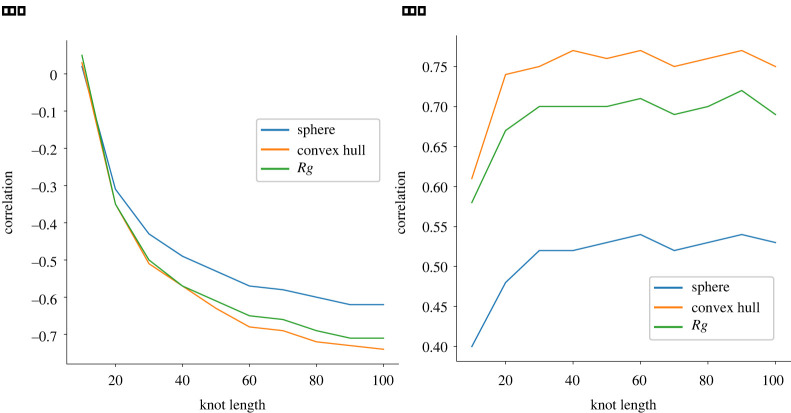
(*a*) The correlation between I(K) and the volume of the circumscribing sphere, convex hull and Rg as a function of knot length. (*b*) The correlation between the length of the maximal bar M(K) in the Vietoris–Rips barcode and the same quantities as above. (Online version in colour.)

We also report the existence of an increasingly positive correlation between M(K) (as displayed in figure 7*b*) and the volume of the convex hull. Other correlations between e.g. #B(K) and the convex hull’s volumes or Rg are marginally weaker than what we observe for I(K), and we did not include this data in this manuscript, referring to the full dataset in [33]. We partitioned our dataset in two, and verified the robustness of the previous analysis by comparing the results obtained on each part.

Interestingly, unlike in the case of the ACN, we do not find any significant correlation between either the curvature or torsion of the knots and the integral I. This should be compared with other more ad hoc approaches, such as [34–36].

**Figure 8.**
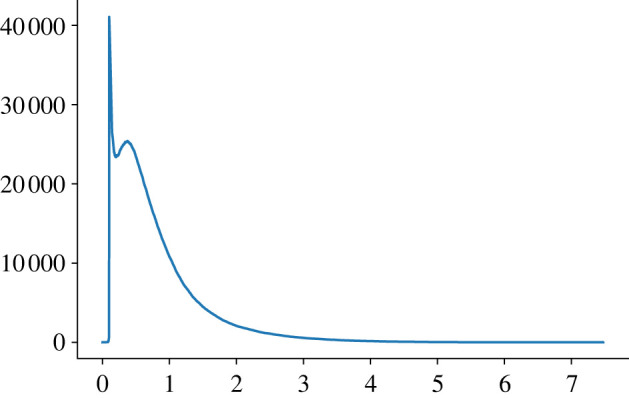
The sum of 1000 Betti curves for random unknots of length 200. This overall shape is present for all knot types considered. The average curve can be obtained simply by dividing by 1000 pointwise. (Online version in colour.)

We also look at the sum of all Betti curves for each length and/or knot type. It turns out that the overall shape of this ‘cumulative’ curve is the same, regardless of the knot type. One example of such a curve is shown in figure 8 for the case of unknots of length 200. Note that the parameter t used by Ripser is the diameter of the points’ neighbourhoods, rather than the radius, which has the effect of stretching the domain’s length by two in figure 8.

**Figure 9.**
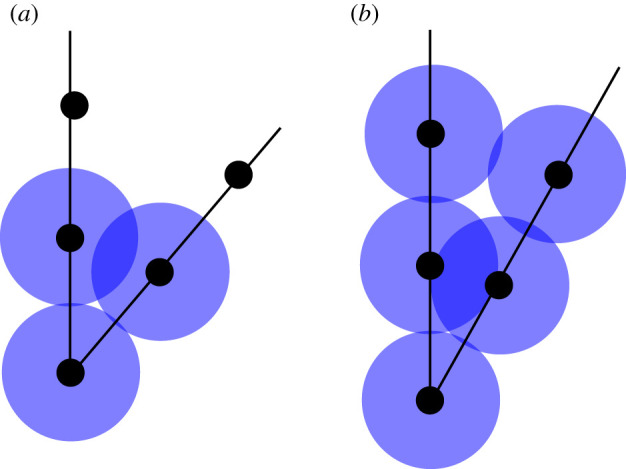
(*a*) A short-lived homology class in the Čech complex of P(K); these appear for angles smaller than π/3, when the vertex point of a PL knot embedding K, together with the two adjacent points in P(K), have neighbourhoods that only intersect in pairs for a small range of radii. When all three disks intersect, the length-three 1-cycle they generate in the Čech complex is capped off by a 2-simplex. (*b*) The analogous situation for the Vietoris–Rips complex, where four vertices are needed to create a short-lived class. In this case, the angle has to be smaller than $\arccos(\frac{3}{4})$. (Online version in colour.)

It is interesting to point out certain clear characteristics of these cumulative functions, which are present for each choice of knot type and length. The leftmost spike indicates the presence of a large amount of rather short-lived homology classes that appear right after t=0.1. This is due to the fact that two consecutive segments in a PL knot embedding whose internal angle is less than or equal to $\arccos\left(\frac{3}{4}\right)∼41.4^{\circ }$ will produce such a class, as shown in figure 9. Therefore, the presence of this spike can be regarded as a consequence of our choice to use the Vietoris–Rips complex as an approximation of a knot’s neighbourhood. However, as pointed out in figure 9*a*, a similar (potentially wider and higher) spike would have appeared if we had chosen the Čech complex instead. Incidentally, the value of the maximum attained is therefore related to the average number of small angles present in the embeddings. It is possible to remove this spike by simply ignoring all short-lived bars appearing right after t=0.1.

Similarly, the value of the second maximum might be of interest, as it appears to be related to the average distance between the edges of PL embeddings in the given knot type.

**Figure 10.**
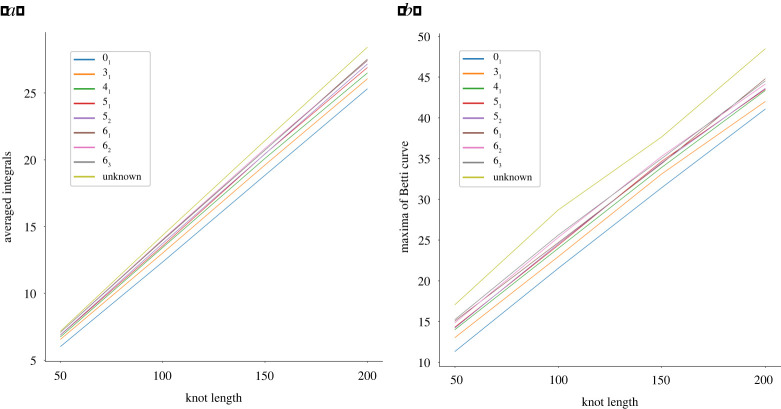
(*a*) Average of the integrals of the Betti curves for each knot type as a function of length. (*b*) Average of the maxima of the Betti curves (figure 8) for each knot type as a function of length. (Online version in colour.)

**Figure 11.**
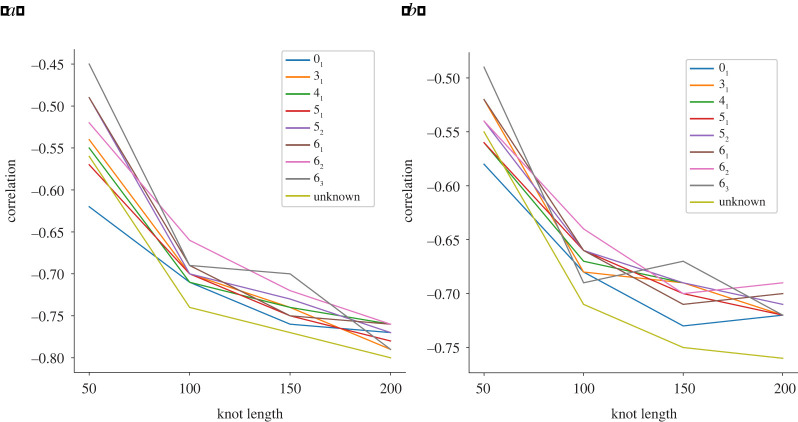
Here, we focus on the correlations between I(K) and the volume of the convex hull (*a*) and Rg (*b*). We are splitting the correlations according to the various knot types considered. (Online version in colour.)

**Figure 12.**
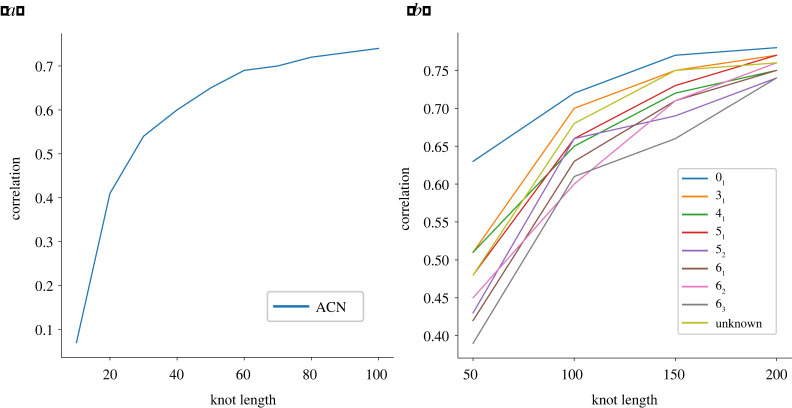
Correlation functions between the average crossing number and I(K). In (*a*), we consider all knots (with lengths ranging from 10 to 100), while in (*b*), we are splitting among the various knot types considered (with lengths ranging from 50 to 200). (Online version in colour.)

We display the values of the average integral and the maximum of the average Betti curves for the various knot types considered as a function of length in figure 10. In both cases, there appears to be an almost perfect linear relation. Furthermore, unlike what can be observed in figures 11 and 12, here we have a clear division into knot types, with values increasing monotonically with the knot type’s topological complexity. A possible explanation for the higher (inverse) correlation between knot length and volume/$R_{g}$ displayed in figure 11 is that many of these rather long knots might be composite and hence localized.

## 6. Deviation from ideality

In this final section, we use the tools considered so far to introduce a naive way of quantifying ‘how much’ a given knot deviates from being ideal. The key idea follows from remark 2.3: we can use the fact that the PH of ideal knots has a predictable behaviour to quantify the dissimilarity of the given knot from ideal knot embeddings. Note that our measure will quantify the difference of a given embedding to all possible ideal embeddings, rather than just those belonging to the same knot type.

Denote by $S(K)=\max\{t\geq 0|\beta_{1}(K)\neq 0\}$, and let ε be a small positive real number. Call $f_{R,\varepsilon }:[0,R]⟶R_{\geq 0}$ the unique function obtained by considering the linear function taking value 1 on 0, and value 0 on R−ε, and defined to be identically 0 after R−ε. Then define

6.1 $$\delta_{\varepsilon }(K)=\frac{1}{S(K)}\int_{0}^{S(K)}f_{S(K),\varepsilon }(t)\cdot \max\{\beta_{1}(K)(t)-1,0\}\;\text{d}t.$$

We claim that $\delta_{\varepsilon }$ defines a sensible quantification of the ‘distance’ between the given embedding and ideal ones. Let us examine the various components of equation (6.1) to substantiate the claim. Recall from remark 2.3 that, for an ideal embedding $K_{I}$ of a knot, the function $\beta_{1}(K_{I})$ takes the value 1 until $t=\frac{1}{2}$, where it jumps to m(K), indicating the appearance of m(K) bars. These may not be the last bars appearing, but it is reasonable to assume that any further bar will be short-lived. This is because, by definition, the self-touching solid torus $\nu_{\text{IR}(K)}(K)$ ‘occupies’ most of the volume surrounding the knot.

As we are modelling a knot, the value of $\beta_{1}(K)$ will usually be at least 1 on the interval [0,S(K)]; we thus calibrate for this information by considering $\max\{\beta_{1}(K)(t)-1,0\}$. The role of the function $f_{R,\varepsilon }$ is to preserve the contribution of bars appearing early (e.g. for small values of the diameter), and to erase the contribution of bars appearing towards the end of the support of $\beta_{1}(K)$. Of course, this is not the only possible choice of a function with this property, but it is definitely one of the simplest. The value of ε acts as a cut-off, meaning that all bars born after R−ε will not contribute to $\delta_{\varepsilon }$.

It can be checked that as expected, for sufficiently small choices of the ε threshold, for the trefoils in figure 4 we have (from left to right) $0<\delta_{\varepsilon }(T_{1})<\delta_{\varepsilon }(T_{2})<\delta_{\varepsilon }(T_{3})$.

## Data Availability

The generated data and code are available at https://github.com/agnesedaniele/knot-confinement-and-PH.
